# Difference in Age- and Sex-adjusted Prevalence of Diseases between Employees and Nonemployees with Health Insurance in Japan

**DOI:** 10.31662/jmaj.2024-0281

**Published:** 2025-02-14

**Authors:** Masakazu Kohda, Akira Okada, Hideo Yasunaga

**Affiliations:** 1 DeSC Healthcare, Inc., Tokyo, Japan; 2 Department of Prevention of Diabetes and Lifestyle-Related Diseases, Graduate School of Medicine, The University of Tokyo, Tokyo, Japan; 3 Department of Clinical Epidemiology and Health Economics, The University of Tokyo, Tokyo, Japan

**Keywords:** claims database, prevalence, health insurance, epidemiology, Japan

## Abstract

**Introduction:**

Administrative claims data are used in clinical studies. However, individuals insured by different insurance systems have different backgrounds, ages, and disease prevalences. This study aimed to examine the crude and adjusted prevalence of diseases between employee and nonemployee health insurance in Japan.

**Methods:**

We conducted a cross-sectional study using the DeSC database, an administrative claims database covering multiple insurers. We calculated the prevalence of 10 disease categories and 6 specific cancers with and without adjustments for age and sex and compared them between the employee (Kempo) and nonemployee (Kokuho) insurance systems.

**Results:**

We identified 740,217 and 3,312,042 individuals covered by Kempo and Kokuho, respectively. The Kokuho group showed a higher crude prevalence of malignancies, endocrinological diseases, mental disorders, neurological diseases, cardiovascular diseases, and kidney or genitourinary diseases. The adjusted prevalence differed in mental disorders (7.2% vs. 10.6%), neurological diseases (10.5% vs. 14.0%), and gastrointestinal diseases (50.1% vs. 34.1%) between the Kempo and Kokuho groups.

**Conclusions:**

While using administrative claims data, researchers should consider differences in patient backgrounds and disease prevalence among insurance providers.

## Introduction

Administrative claims data are globally, extensively utilized for clinical studies ^(1)^. In Japan, various administrative claims data have been recently established, and an increasing number of studies are using them ^(2)^.

Japan implemented universal healthcare coverage in 1961, and nearly all residents are now enrolled in one of the following public healthcare insurance systems ^(3)^: National Health Insurance (Kokuho) provided by municipalities for the self-employed or unemployed and their dependents under 75 years of age, health insurance (Kempo) provided by corporate health insurance associations for salaried employees in big companies and their dependents under 75 years of age, health insurance provided by the Japan Health Insurance Association for salaried employees in small companies and their dependents below 75 years of age, Mutual Aid Association Health Insurance for public servants, and the Advanced Elderly Medical Service System for individuals aged ≥75 years. Approximately 28.1, 28.4, 40.3, 8.7, and 18.4 million people were enrolled in each program in 2021 ^(3)^.

There are several differences in the backgrounds of the groups covered by these insurance systems. Those insured under Kempo potentially have stable jobs as full-time employees in big companies and are relatively financially stable, whereas those insured under Kokuho include self-employed, freelancers, part-time workers, the unemployed, and students―some of whom do not have regular income. Those insured under Kempo are mostly working-age people under 65 years, whereas those insured under Kokuho include several age groups and a particularly large number of older and retired individuals.

The Japanese government collects administrative claims data from all insurers to create the National Database of Health Insurance Claims and Specific Health Checkups of Japan (NDB) ^(3)^. Moreover, several private companies independently collect administrative claims data from single or multiple insurers to create original databases and provide these data to researchers commercially ^(2), (4)^. Thus, researchers must carefully select data sources from the available administrative claims databases to select a suitable source for their study.

Studies have reported the influence of socioeconomic status on health outcomes, including income and insurance status^(5), (6), (7)^. In a previous Japanese study, the employment status was associated with participation in breast cancer screening among women ^(8)^. Therefore, the insurance types in Japan may work as a proxy for socioeconomic status. Because of the differences in insured participants, health status may differ between the Kempo and Kokuho groups in Japan. To our knowledge, no previous study has examined whether disease prevalence differs between the Kempo and Kokuho populations. This study aimed to assess the age- and sex-adjusted prevalence of disease among individuals in the Kempo and Kokuho groups.

## Materials and Methods

### Data source

This study utilized administrative claims data from the DeSC database provided by DeSC Healthcare, Inc., Tokyo, Japan (https://desc-hc.co.jp/company). The DeSC database contains healthcare administrative claims data of approximately 13,000,000 individuals whose healthcare insurance is administered by Kokuho, Kempo, or the Advanced Elderly Medical Service System ^(4)^. We used data of individuals who were covered by Kokuho or Kempo. The claims data included medical and dental diagnoses, which were recorded using the International Classification of Diseases 10^th^ Revision (ICD-10) codes and Japanese free text.

### Study design, population, and variables

We conducted a cross-sectional study to compare disease prevalence between the Kempo and Kokuho groups between April 2020 and March 2021. We extracted data on sex, age (as of April 2020), and ICD-10-based diagnoses of infectious diseases (A00-B99), malignancies (C00-C97), endocrinological diseases (E00-E90), mental disorders (F00-F99), neurological diseases (G00-G99), eye and ear diseases (H00-H59), cardiovascular diseases (I00-I99), respiratory diseases (J00-J99), gastrointestinal diseases (K00-K93), and kidney or genitourinary diseases (N00-N99). Among the malignancies, we selected the stomach (C16), colon (C18), bronchus and lung (C34), breast (C50), uterine cervix (C53), and prostate (C61) cancers. Only those with records of diagnoses for ≥2 months during the 1-year study period were included.

### Statistical analysis

We compared the age and sex distributions of the Kempo and Kokuho groups in the DeSC database. Additionally, the age and sex distributions of individuals included in the Kempo and Kokuho groups in the DeSC database were compared with those in Japan as a whole. Data on the Kempo and Kokuho groups of whole Japan were obtained from a report by the National Federation of Health Insurance Societies ^(9)^ and the Ministry of Health, Labour and Welfare (MHLW), respectively ^(10)^. Furthermore, we obtained information on the mean age of each group from another report from the MHLW ^(11)^.

We calculated the crude prevalence of the diseases between April 2020 and March 2021 and compared them between the Kempo and Kokuho groups. The disease prevalence was reported as the proportion of individuals with each disease among the total number of individuals, stratified by 5-year age categories, sex, and insurance type (Kempo or Kokuho).

Moreover, we calculated the age- and sex-adjusted prevalence of each disease and compared them between the Kempo and Kokuho groups. Age and sex adjustments are frequently employed to estimate the true prevalence within a population, assuming a specific age and sex distribution ^(12), (13), (14)^. Herein, individuals in the DeSC database were the study population, and the whole Japanese population in 2020 was the standard population; data on the whole Japanese population were derived from the Population Census ^(15)^. First, the age- and sex-specific proportions for each age and sex group in the study population were multiplied by the corresponding weight in the standard population. All products were added for obtaining the age- and sex-adjusted prevalences. To calculate cancer prevalence, we limited the study population to individuals aged ≥40 years for stomach, colon, or lung cancer; women aged ≥20 years for breast cancer; women aged ≥30 years for cervical cancer; and men aged ≥45 years for prostate cancer.

Because of the large sample size, standardized mean differences were computed to assess the difference in covariates between the two groups. An absolute standardized mean difference of <0.1 was considered to indicate a negligible imbalance between the groups ^(16)^. Statistical analyses were performed using Stata version 18 (StataCorp, College Station, TX, USA).

### Ethical statement

This study protocol was approved by the Institutional Review Board of the Graduate School of Medicine at the University of Tokyo (approval number: 2021010NI). Owing to the use of anonymized data, the requirement for informed consent was waived. The study adhered to the ethical principles per the Declaration of Helsinki.

## Results

### Population

We identified 4,052,259 individuals aged <75 years in the DeSC database, including 740,217 and 3,312,042 covered by the Kokuho and Kempo groups. Table 1 presents the age and sex distributions of these two groups. Individuals covered by Kempo were younger than those covered by Kokuho. The comparison of age and sex distributions among individuals included in the Kempo and Kokuho groups in the DeSC database with those in Japan as a whole are presented in Supplementary Figs. S1 and S2. The mean ages of individuals in the Kempo and Kokuho groups in the DeSC database were 39.4 and 54.6 years, respectively, whereas those of the whole population in these groups were 35.2 and 53.6 years, respectively. The proportions of men among individuals covered by the Kempo and Kokuho groups in the DeSC database were 54.4% and 48.8%, respectively, whereas those of the whole population in these groups were 52.4% and 48.1%, respectively.

**Table 1.** Age and Sex Distributions in the Kempo and Kokuho Groups.

	Employee insurance (Kempo)	Nonemployee insurance (Kokuho)	Standardized mean difference
	(N = 740,217)	(N = 3,312,042)	
Male	402,376 (54.4%)	1,616,526 (48.8%)	−0.112
Age (years)			
0-4	16,888 (2.3%)	24,336 (0.7%)	−0.132
5-9	40,313 (5.4%)	70,975 (2.1%)	−0.174
10-14	42,618 (5.8%)	86,262 (2.6%)	−0.160
15-19	44,768 (6.0%)	96,423 (2.9%)	−0.151
20-24	47,994 (6.5%)	103,664 (3.1%)	−0.160
25-29	41,750 (5.6%)	98,092 (3.0%)	−0.128
30-34	45,981 (6.2%)	104,111 (3.1%)	−0.148
35-39	54,825 (7.4%)	134,070 (4.0%)	−0.147
40-44	62,651 (8.5%)	161,838 (4.9%)	−0.144
45-49	77,164 (10.4%)	201,677 (6.1%)	−0.157
50-54	85,474 (11.5%)	223,506 (6.7%)	−0.167
55-59	75,132 (10.1%)	206,144 (6.2%)	−0.143
60-64	48,995 (6.6%)	265,457 (8.0%)	0.054
65-69	29,651 (4.0%)	532,098 (16.1%)	0.411
70-74	26,013 (3.5%)	1,003,389 (30.3%)	0.766

### Prevalence of major diseases according to insurance type

Supplementary Fig. S3 presents the crude prevalence of the disease categories stratified by age, sex, and insurance type. Infectious and respiratory diseases were more common in young and older individuals than in middle-aged individuals. The prevalence of the malignancies, endocrinological diseases, neurological diseases, mental disorders, and kidney or genitourinary diseases increased with age. The prevalence of eye and gastrointestinal diseases also increased with age, but both decreased in those aged 15-30 years. Sex differences were evident in the malignancies, mental disorders, and kidney or genitourinary diseases. While examining the insurance types, gastrointestinal diseases were more common among individuals covered by Kempo than among those covered by Kokuho, whereas mental disorders and neurological diseases were more prevalent among those covered by Kokuho than among those covered by Kempo.

Table 2 shows the disease prevalence stratified by the insurance type before and after adjusting for age and sex. Without adjustments, the crude prevalences of the malignancies, endocrinological diseases, mental disorders, neurological diseases, cardiovascular diseases, and kidney or genitourinary diseases were higher in the Kokuho group than in the Kempo group (absolute standardized mean difference > 0.1). However, after age and sex adjustments, the prevalences were not significantly different between the groups for malignancies or endocrinological, cardiovascular, kidney, or genitourinary diseases (absolute standardized mean difference < 0.1). Mental disorders and neurological diseases were more frequent in the Kokuho group than in the Kempo group, even after the adjustment (absolute standardized mean difference > 0.1). The prevalence of gastrointestinal diseases was higher in the Kempo group than in the Kokuho group after adjusting for age and sex (absolute standardized mean difference > 0.1).

**Table 2.** Disease Prevalence before and after Age and Sex Adjustment, Stratified by Insurance Type.

Disease categories	Crude prevalence			Age- and sex-adjusted prevalence		
	Kempo	Kokuho	SMD	Kempo	Kokuho	SMD
Infectious diseases	10.2%	12.8%	0.083	10.9%	10.9%	−0.003
Malignancies	**2.1%**	**4.9%**	**0.155**	2.5%	2.2%	−0.015
Endocrinological diseases	**17.1%**	**33.5%**	**0.383**	19.0%	18.8%	−0.005
Mental disorders	**7.2%**	**11.5%**	**0.148**	**7.2%**	**10.6%**	**0.119**
Neurological diseases	**9.8%**	**19.8%**	**0.284**	**10.5%**	**14.0%**	**0.107**
Eye diseases	17.8%	21.3%	0.090	18.6%	15.4%	−0.085
Cardiovascular diseases	**13.1%**	**31.1%**	**0.445**	14.8%	15.4%	0.016
Respiratory diseases	24.4%	24.9%	0.012	25.9%	24.3%	−0.035
Gastrointestinal diseases	49.0%	45.3%	−0.075	**50.1%**	**34.1%**	**−0.329**
Kidney or genitourinary diseases	**9.9%**	**14.2%**	**0.133**	10.8%	10.2%	−0.022

SMD, standardized mean difference Bold fonts indicate significant differences in prevalence between the Kempo and Kokuho groups.

### Prevalence of specific cancers stratified by insurance type

Supplementary Fig. S4 presents the crude prevalence of specific cancers stratified by age, sex, and insurance type. Regarding age distribution, the prevalence of the stomach, colon, lung, and prostate cancers increased with age, whereas that of cervical cancer peaked between 40 and 50 years and that of breast cancer peaked between 50 and 60 years of age. Both insurance types showed the same peak age for most cancers, whereas the Kempo group had a slightly higher crude prevalence of each cancer than the Kokuho group.

Table 3 shows the prevalence of specific cancers stratified by insurance type before and after the adjustment for age and sex. Neither crude nor adjusted prevalence was significantly different between the Kempo and Kokuho groups for any cancer (absolute standardized mean differences < 0.1).

**Table 3.** Prevalence of Specific Cancers before and after Age and Sex Adjustment, Stratified by Insurance Type.

Cancer	Crude prevalence			Age- and sex-adjusted prevalence		
	Kempo	Kokuho	SMD	Kempo	Kokuho	SMD
Stomach	0.3%	0.7%	0.057	0.4%	0.4%	−0.009
Colon	0.3%	0.8%	0.063	0.4%	0.5%	0.006
Bronchus and lungs	0.2%	0.6%	0.058	0.3%	0.3%	0.005
Breast	1.5%	1.9%	0.029	1.5%	1.2%	−0.026
Uterine cervix	0.2%	0.2%	0.006	0.2%	0.2%	0.009
Prostate	0.6%	1.6%	0.088	1.0%	0.7%	−0.030

SMD, standardized mean difference

## Discussion

In this analysis of the administrative claims database, the Kokuho group showed higher crude prevalences of the malignancies, endocrinological diseases, mental disorders, neurological diseases, cardiovascular diseases, and kidney or genitourinary diseases than the Kempo group. However, after adjusting for age and sex, the prevalence of mental disorders and neurological diseases was higher in the Kokuho group than in the Kempo group. The adjusted prevalence of gastrointestinal diseases was higher in the Kempo group than in the Kokuho group. There was no notable difference in the adjusted prevalence of the overall malignancy or any cancer type between the Kempo and Kokuho groups.

The Kokuho group included a relatively older population, which may have impacted the higher crude prevalence of malignancies, endocrinological diseases, cardiovascular diseases, and kidney or genitourinary diseases observed. The age- and sex-adjusted prevalences of these diseases were not significantly different between the Kempo and Kokuho groups, highlighting the importance of adjusting for age and sex when evaluating disease prevalence across different populations as unadjusted comparisons can lead to biased estimates.

Herein, the Kokuho group showed a higher prevalence of mental disorders and neurological diseases, even after adjusting for age and sex; this suggests that several factors, other than age and sex, may affect this difference. However, only a few previous studies have examined the difference in the prevalence of mental disorders between employees and nonemployees. A previous cross-sectional study suggested that insurance provided by the workplace was associated with a lower prevalence of antepartum depression than insurance provided by parent or Medicaid among low-income women in the US ^(17)^. A previous cohort study demonstrated that individuals with mental disorders were more likely to quit their jobs than those without them, leading to a transition to nonemployee-based insurance ^(18), (19)^. Herein, the high prevalence of mental disorders in the Kokuho group could also be explained by the transition of individuals with mental disorders from the Kempo to Kokuho group.

Additionally, this study reported that the prevalence of gastrointestinal diseases was higher in employees than in nonemployees, even after adjusting for age and sex; the reason for this finding remains unclear and should be investigated in the future; however, nonemployees with mild gastrointestinal symptoms may not be able to access healthcare services.

Nevertheless, this study has several limitations. The use of claims data may limit the validity of diagnoses or lead to failure in disease severity assessment. Although cancer reimbursements have high specificity (> 95%) ^(20)^, there may still be some misclassification. Moreover, although we adjusted for age and sex, other potential confounders that could have influenced the observed prevalence, such as lifestyle factors, were not accounted for.

In conclusion, the Kokuho group showed a higher age- and sex-adjusted prevalence of mental disorders and neurological diseases, whereas the Kempo group showed a high age- and sex-adjusted prevalence of gastrointestinal diseases when differences in disease prevalence between Japanese insured populations were examined. While conducting studies using administrative claims data, researchers should consider differences in population backgrounds and disease prevalence between insurers.

## Article Information

### Conflicts of Interest

Masakazu Kohda is an employee of DeSC Healthcare, Inc. Akira Okada is a member of the Department of Prevention of Diabetes and Lifestyle-related Diseases, which is a cooperative program between the University of Tokyo and the Asahi Mutual Life Insurance Company.

### Author Contributions

MK and AO performed data collection, statistical analysis, and wrote the first draft. HY designed the research, revised the first draft, and supervised manuscript preparation. All authors have read and approved the final manuscript.

### Approval by Institutional Review Board (IRB)

This study protocol was approved by the Institutional Review Board of the Graduate School of Medicine at the University of Tokyo (2021010NI), and the study was conducted in accordance with the principles of the Helsinki Declaration.

### Informed Consent

Owing to the retrospective nature of this study, which utilized a commercially available database, the requirement for informed consent was waived.

## Supplement

Supplementary Fig. S1 Proportion of individuals covered by Kempo in the DeSC database and in Japan stratified by age and sex

Supplementary Fig. S2 Proportion of individuals covered by Kokuho in the DeSC database and in Japan stratified by age and sex

Supplementary Fig. S3 Crude prevalence of the disease categories stratified by age, sex, and insurance type

Supplementary Fig. S4 Crude prevalence of specific cancers stratified by age, sex, and insurance type
