# Supplementary figures and images for: Difference in Age- and Sex-adjusted Prevalence of Diseases between Employees and Nonemployees with Health Insurance in Japan
Source: JMA J. 2025 Feb 14;8(2):411–6. doi: 10.31662/jmaj.2024-0281 (PMC12095125; doi:10.31662/jmaj.2024-0281)

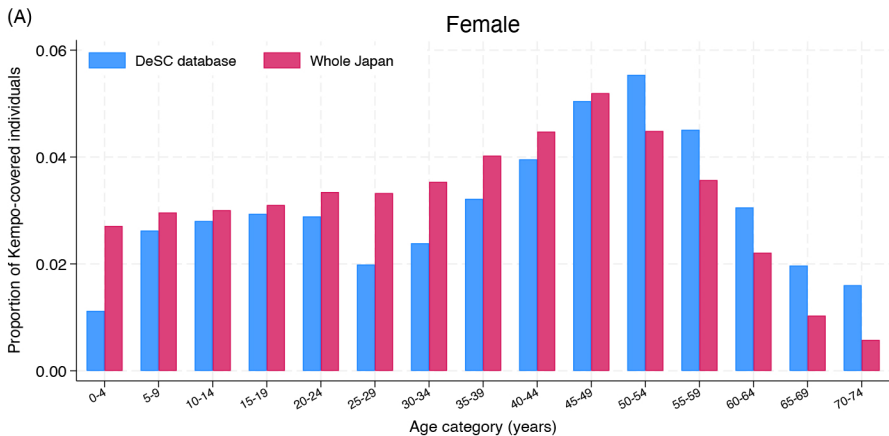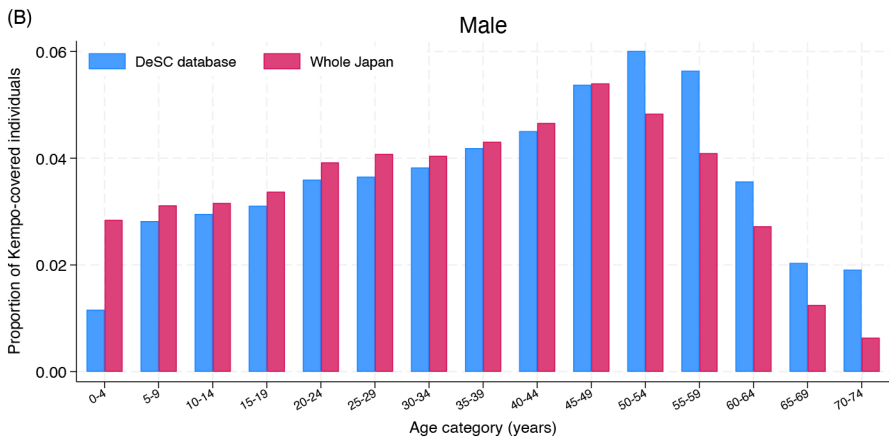

Supplement: Supplementary Fig. S1 — Proportion of individuals covered by Kempo in the DeSC database and in Japan stratified by age and sex [file 2433-3298-8-2-0411-s001.pdf]

(A)

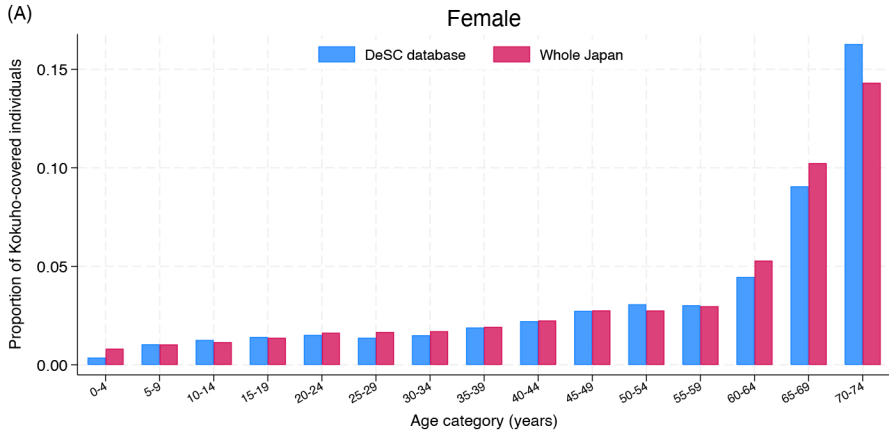

(B)

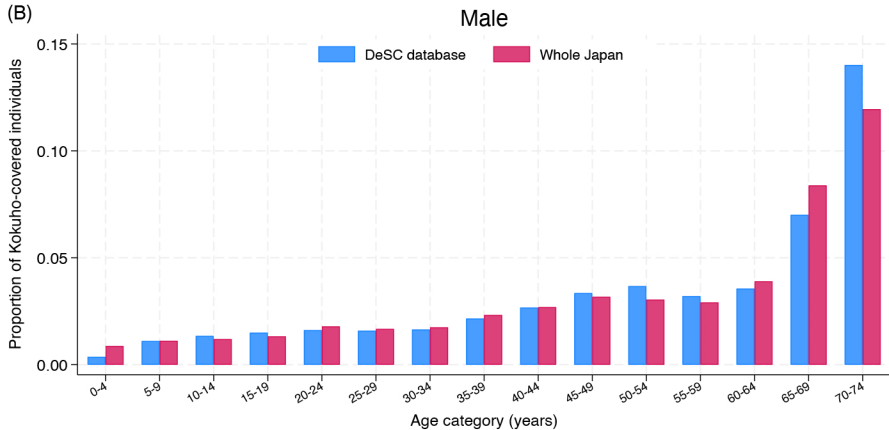

Supplement: Supplementary Fig. S2 — Proportion of individuals covered by Kokuho in the DeSC database and in Japan stratified by age and sex [file 2433-3298-8-2-0411-s002.pdf]

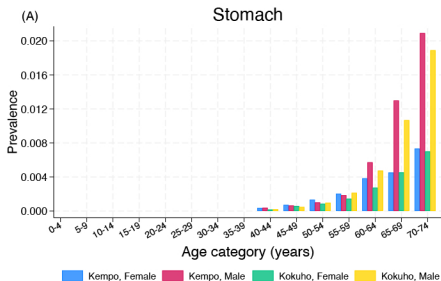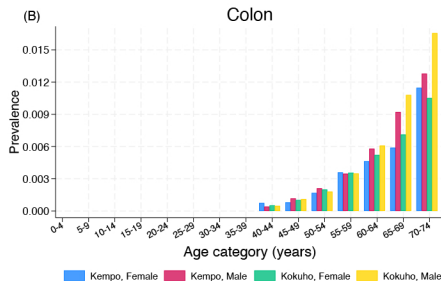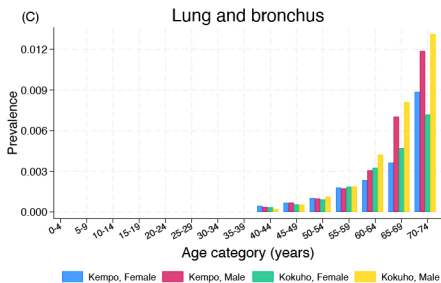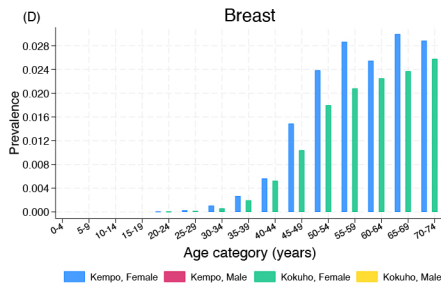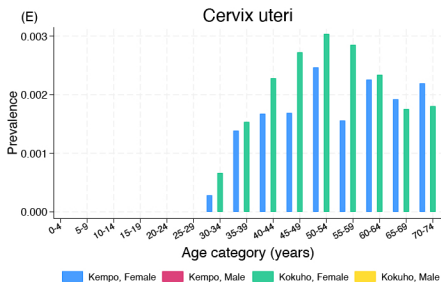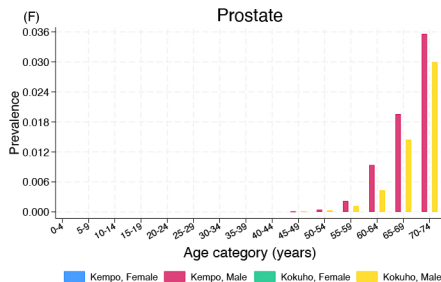

Supplement: Supplementary Fig. S4 — Crude prevalence of specific cancers stratified by age, sex, and insurance type [file 2433-3298-8-2-0411-s004.pdf]
