# Supplementary material for: Difference in Age- and Sex-adjusted Prevalence of Diseases between Employees and Nonemployees with Health Insurance in Japan
Source: JMA J. 2025 Feb 14;8(2):411–6. doi: 10.31662/jmaj.2024-0281 (PMC12095125; doi:10.31662/jmaj.2024-0281)
Supplement: Supplementary Fig. S3 — Crude prevalence of the disease categories stratified by age, sex, and insurance type [file 2433-3298-8-2-0411-s003.pdf]

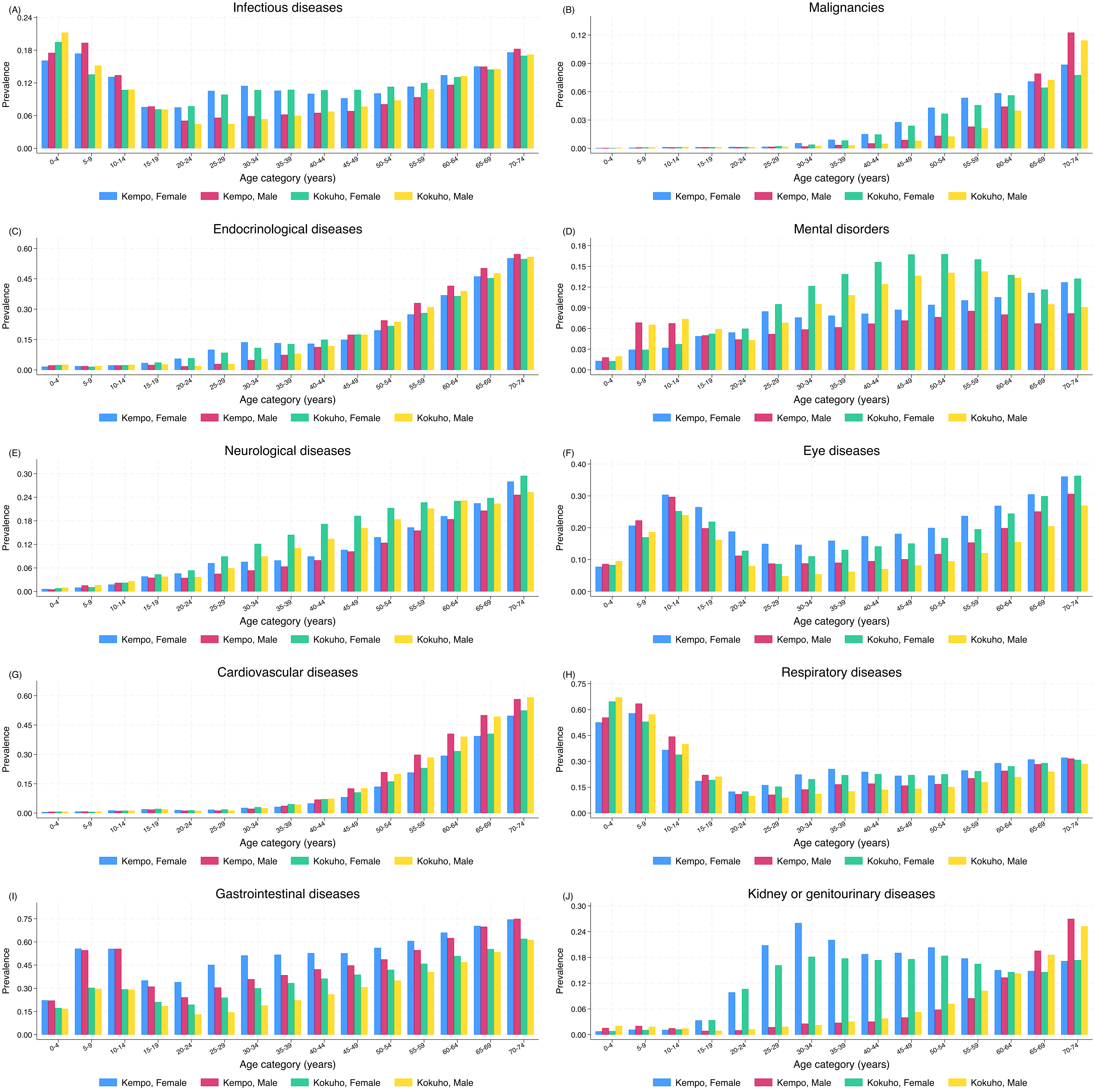
(A)
Infectious diseases
(B)
Malignancies
(C)
Endocrinological diseases
(D)
Mental disorders
(E)
Neurological diseases
(F)
Eye diseases
(G)
Cardiovascular diseases
(H)
Respiratory diseases
(I)
Gastrointestinal diseases
(J)
Kidney or genitourinary diseases
Prevalence
Age category (years)
0-4
5-9
10-14
15-19
20-24
25-29
30-34
35-39
40-44
45-49
50-54
55-59
60-64
65-69
70-74
Kempo, Female
Kempo, Male
Kokuho, Female
Kokuho, Male
